# Research progress in the mechanism of acupuncture regulating microglia in the treatment of Alzheimer’s disease

**DOI:** 10.3389/fnins.2024.1435082

**Published:** 2024-07-31

**Authors:** Jia Liu, Jiaqi Zhou, Chong You, Haonan Xia, Yuling Gao, Yong Liu, Xiaoyang Gong

**Affiliations:** ^1^Department of Rehabilitation Medicine, The First Affiliated Hospital of Dalian Medical University, Dalian, China; ^2^Dalian Medical University College of Integrated Traditional Chinese and Western Medicine, Dalian, China

**Keywords:** acupuncture, Alzheimer’s disease, microglia, mechanism research, neuroinflammation

## Abstract

Alzheimer’s disease (AD) is the most common neurodegenerative disease in the central nervous system, characterized by memory and cognitive dysfunction. Acupuncture is an effective means to alleviate the symptoms of AD. Recent studies have shown that microglia play an important role in the occurrence and development of AD. Acupuncture can regulate the activity of microglia, inhibit neuroinflammation, regulate phagocytosis, and clear Aβ Pathological products such as plaque can protect nerve cells and improve cognitive function in AD patients. This article summarizes the relationship between microglia and AD, as well as the research progress in the mechanism of acupuncture regulating microglia in the treatment of AD. The mechanism of acupuncture regulating microglia in the treatment of AD is mainly reviewed from two aspects: inhibiting neuroinflammatory activity and regulating phagocytic function.

## 1 Background

Alzheimer’s disease (AD) is a neurodegenerative disease in the elderly. With the aging of the population, its prevalence has increased year by year. In the early stage, the main manifestations are memory impairment, cognitive impairment, and even the late loss of life self-care ability. The pathogenesis of AD is numerous, mainly including Amyloid β-protein(Aβ) Mechanisms of abnormal phosphorylation of tau protein, neuroinflammation, metabolic disorders, cholinergic mechanisms, oxidative stress, etc. Neuroinflammation plays a central role in regulating neurogenic changes induced by AD (Sung et al., 2020). Neuroinflammation is also closely related to the accumulation of pathological products such as Aβ and tau proteins. As the main participant in neuroinflammation, microglia are closely related to the occurrence and development of AD, playing an extremely important regulatory role in the pathological process of AD (Nordengen et al., 2019).

Microglia are innate immune cells in the central nervous system (CNS). They play a key regulatory role in neuroinflammation, immune defense, and repair of synaptic plasticity under both physiological and pathological conditions (Shabab et al., 2017). Various stimuli can activate microglia, including immune factors, pathogens, injuries, neurotoxins, and various diseases (Bivona et al., 2023). Microglia have a “double edged sword” effect on AD, involving neurotoxicity or neuroprotective function based on background factors and disease stages (Hickman et al., 2018). In summary, in the early stages of AD, microglia participate in the elimination of pathological products. With the development of disease, the continuous activation of microglia will ultimately lead to a chronic inflammatory state, adversely affecting neuronal survival and causing direct neuronal damage. Therefore, restoring the homeostasis of microglia and consuming the number of microglia by studying the key targets of microglia is considered to be an effective way to treat cognitive dysfunction (Fei Li and Yun, 2021), which is currently a research hotspot.

**Figure 1:**
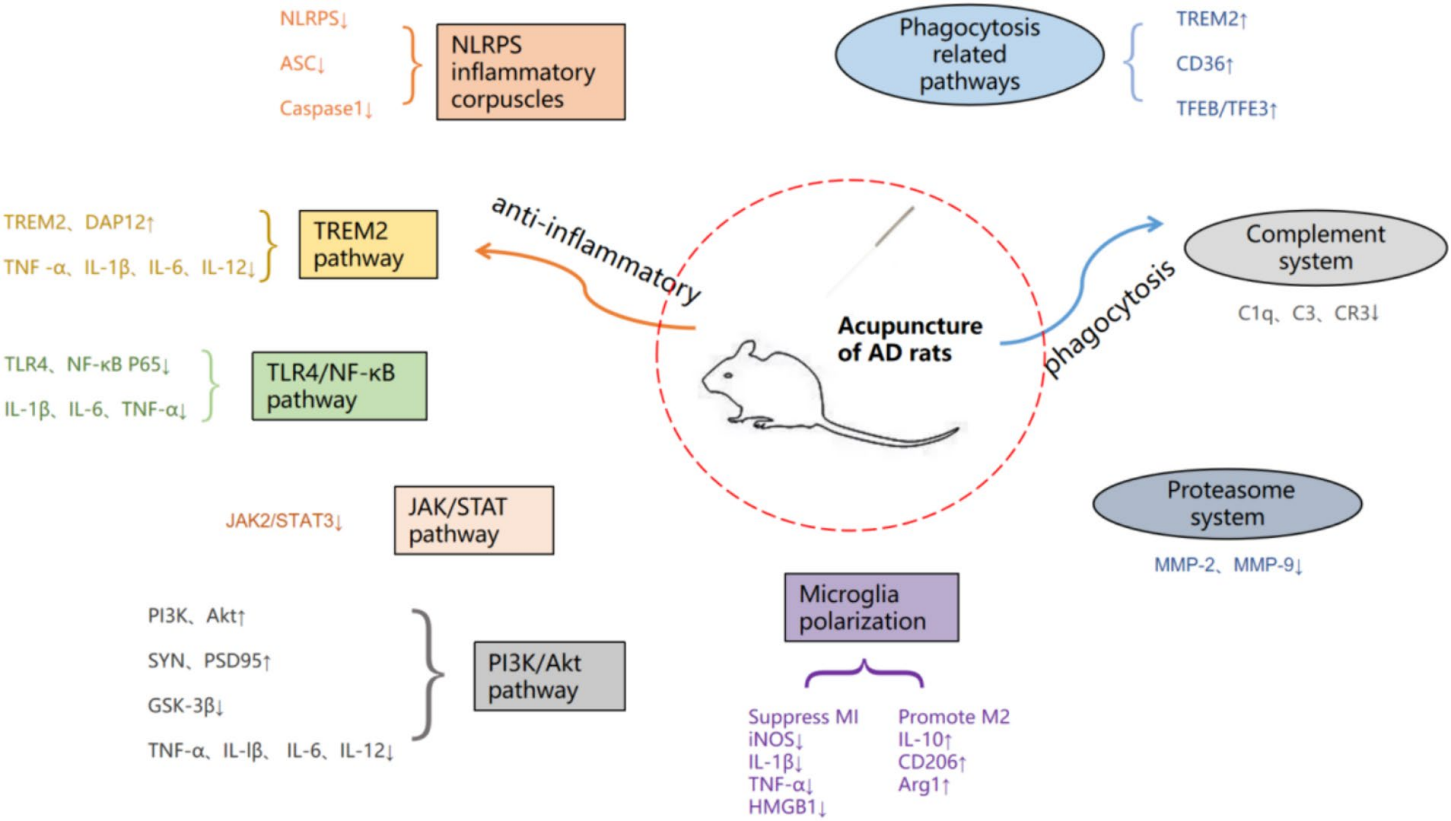
Summary of acupuncture therapy for AD pathway mechanisms.

Acupuncture, as an important method in traditional medicine, can effectively improve the cognitive and living abilities of patients, alleviate symptoms, and reduce adverse reactions to acupuncture, which is beneficial for long-term treatment of patients with chronic diseases. Modern studies have shown that acupuncture can ameliorate the cognitive decline associated with AD by modulating microglia neuroinflammatory and phagocytic functions and thereby improving the cognitive decline associated with AD (Xie et al., 2021; Hong et al., 2022). However, the specific mechanism is not yet comprehensive, therefore, in this paper, by searching the China Knowledge Network (CNKI), Wanfang Data Service Platform, and Pub Med databases with “Acupuncture” “Alzheimer’s disease” “Microglia” “Inflammation” as the search terms, the search time is from 2013 to now (July 1, 2024), nearly 11 years of research has been done. The basic researches on the modulation of microglia by acupuncture through neuroinflammation and phagocytosis in the treatment of AD in the past 11 years were summarized and analyzed (Figure 1), with a view to further elucidating the mechanism of action of acupuncture in the treatment of AD.

## 2 Characteristics of microglia

The nervous system consists of two major types of cells, namely glial cells and neurons. The glial cells of CNS mainly include astrocytes, microglia, oligodendrocytes, choroid plexus cells, etc., and their number is 10 to 50 times that of neurons. Among them, microglia account for about 20% of the total number of glial cells (Wang M, et al., 2022). Microglia are the only neural cells derived from the mesoderm and are specialized macrophages that serve as resident immune cells in the CNS to maintain brain homeostasis (Badimon et al., 2020). microglia perform immune functions in the nervous system, mainly to remove harmful substances, repair damage, and protect various types of neurons in the CNS. Neurons can generate and transmit action potentials over long distances through synaptic connections, while microglia only participate in local synaptic interactions, sensing the electrical activity of axons by ingesting calcium ions, and maintaining internal environment stability through the uptake of neurotransmitters and ions (Cornell et al., 2022). Microglia enter the developing brain long before neurogenesis, neuronal migration, myelination of various brain regions, and glial formation of regional neuroepithelial cells (Menassa and Gomez-Nicola, 2018). Microglia are believed to play a passive or supportive role in neurons, and are now known to be very active in the CNS, and are associated with other brain intrinsic immune cells, neuronal circuit development, synaptic pruning, myelin turnover, and neuronal excitability control.

Microglia are a group of highly heterogeneous cells that can polarize from a resting state (M0 microglia) into two main activated phenotypes, the classic activated M1 microglia and the replacement activated M2 microglia, which involve monitoring, proinflammatory, and anti-inflammatory responses (Wendimu and Hooks, 2022). The markers of M1 and M2 are arginase 1 (Arg1) and inducible nitric oxide synthase (iNOS), respectively. The M1 phenotype is responsible for eliminating pathological invaders, providing a proinflammatory state, and releasing high levels of proinflammatory cytokines and chemokines (such as IL-1β、IL-6, IL-12 and TNF-α) And low levels of anti-inflammatory mediators (such as IL-10) play a key role. In contrast, M2 phenotypic distribution is associated with anti-inflammatory status, with high levels of IL-10 and Transforming growth factor β (TGF-β), Insulin like growth factor 1 (IGF-1) and brain derived neurotrophic factor (BDNF) are beneficial for protecting neurons and tissue repair (Hao et al., 2023). After being stimulated to a certain extent, microglia will be activated into an M1 pro-inflammatory phenotype or an M2 anti-inflammatory phenotype, eliminating microorganisms, dead cells, redundant synapses, protein aggregates, and other particles and soluble antigens that may endanger the CNS (Colonna and Butovsky, 2017; Wolf et al., 2017). M1 and M2 are not two independent activation states, but represent a series of continuous activation phenotypes, and different phenotypic markers can coexist, indicating the existence of many intermediate phenotypes (Song and Suk, 2017). In summary, the role of microglia in healthy and pathological brains and the changes between various phenotypic states depend on the mechanism involved, the time of injury, and the regulatory signaling molecules involved (Zhang, 2019).

In addition, under normal physiological conditions, the number and function of microglia are strictly controlled by the local microenvironment and interactions with surrounding cells. Microglia can be activated by infection or injury in brain tissue, presenting as large and round amoeba-like cells that quickly reach the injured site to phagocytose apoptotic and damaged cells, or release toxic factors to directly destroy infected cells. Relevant studies have shown that the toxic factors released by microglia can also damage surrounding normal tissues, and when microglia are abnormally activated, they can lead to neurotoxicity, which can lead to neurodegenerative diseases (Subhramanyam et al., 2019). Studies have shown that microglia regulate neurons during the life cycle, and in young healthy brains, microglia actively regulate neurogenesis. The activity of microglia decreases with age, and long-term expression of proinflammatory factors and display impaired phagocytic activity, accompanied by the release of neurotoxic cytokines, impairing neurogenesis and synaptic integrity, Resulting in neuronal loss and cognitive decline in AD (al-Onaizi et al., 2020). Mice with depletion of microglia exhibit deficits in learning and memory formation (Wei and Li, 2022).

## 3 Relationship between AD and microglia

### 3.1 Aβ and microglia

The proliferation and activation of microglia are concentrated near amyloid plaques, which are the main characteristics of AD microglia (Hansen et al., 2018). Expression of microglia surface receptors and inflammatory markers (including CD36, CD14, CD11c, MHC-II, and iNOS), as well as M1 phenotypic indicators (such as IL-1β, MCP-1, MIP-1, IL-1, TNF, and IL-6) were found in the AD mouse model. The expression of chemokine receptors (CCL-2, CCL-3, and CCL-4) means that microglia are in an activated state in AD (Kamphuis et al., 2016; Martin et al., 2017). Activated microglia in AD play a “scavenger” role, such as clearing Aβ, Pathological products such as redundant synapses can delay the progression of AD. Triggering Receptor Expressed On Myeloid Cells 2 (TREM2) is an important innate immune receptor on the surface of microglia, which is greatly associated with the occurrence of AD (Zhong et al., 2019). Studies have confirmed that TREM2 can activate the downstream spleen tyrosine kinase (SYK) signal pathway to phosphorylate it, thereby promoting the response of microglia to Aβ (Schlepckow et al., 2020). Phagocytosis of plaques while abnormal mutations in TREM2 can cause microglia to clear Aβ (Zhao et al., 2018). In addition, Aβ is associated with other microglia receptors such as Toll-like receptor (TLR), CD36, class A1 scavenger receptor (SR-A1), and receptor for advanced glycation end (RAGE) also mediates the phagocytosis of Aβ by microglia (Yu and Ye, 2015). Microglial complement receptor 3 (CR3) can reduce Aβ levels in the brain of APP transgenic mice. In addition, published studies have shown that microglia can also play a role in clearing Aβ by regulating proteases such as enkephalinase, insulin degrading enzyme, matrix metalloproteinase 2 (MMP2), MMP9, and tissue plasminogen activator (tPA/plasmin; Corraliza-Gómez et al., 2022).

Aβ is associated with microglia, not only phagocytosis, but also inflammation. This inflammatory relationship exhibits different anti-inflammatory and proinflammatory responses in the early and late stages of AD, respectively. In the early stages of disease progression, microglia mainly exhibit the M2 anti-inflammatory phenotype, and the release of anti-inflammatory cytokines can promote the polarization of microglia from M1 type to M2 (Tang and Le, 2016; Tang et al., 2019). Activated microglia can phagocytose cell fragments and release IL-4, IL-10, and TGF-β Anti-inflammatory factors such as VEGF and BDNF can play an anti-inflammatory role while nourishing neurons and promoting the repair of the blood brain barrier (Kang et al., 2020). On the contrary, with the development of AD, continuously activated microglia will lead to a chronic inflammatory state and promote the deterioration of the disease. Studies have shown that in the late stage of AD, microglia mainly have the M1 phenotype, accounting for a large proportion, by inducing the excretion of neurotoxic factors IL-1β, TNF-α, NO induces inflammatory reactions (Li et al., 2018). Research has confirmed that TNF-α, IL-1β, IL-6 is found in both serum and brain tissue of AD patients is increased levels of pro-inflammatory cytokines (Yongkang et al., 2021). The occurrence of this proinflammatory state is related to Aβ activation of small glial cells associated with NLRP3 inflammatory bodies, which can promote downstream IL-1β and IL-18 expression and release (Hanslik and Ulland, 2020). Persistent inflammatory reactions may be the cause of tau disease, synaptic toxicity and dysfunction, as well as neuronal damage (Andronie-Cioara et al., 2023). Proinflammatory cytokines can stimulate the expression of β secretory enzymes increases the processing of amyloid precursor protein (APP), ultimately leading to Aβ (Cheng et al., 2020). Activation of NLRP3 inflammatory corpuscles can also reduce the phagocytic function of microglia and increase Aβ accumulation (Mata Martínez et al., 2022). With the progress of AD, chronically activated microglia gradually lose clearance Aβ ability and efficiency (Li et al., 2018), while continuously producing proinflammatory and neurotoxic factors, leading to neurodegeneration.

### 3.2 Tau protein and microglia

In healthy brains, tau protein is a rich microtubule binding protein that is mainly expressed in the axons of mature neurons, participates in microtubule formation, maintains microtubule stability, and plays an important role in axonal transport (Chi et al., 2018). Although tau protein phosphorylation is necessary for normal physiological function (Chidambaram et al., 2020), the neurofibrillar tangle (NFT) formed by tau protein hyperphosphorylation is an important pathological marker of AD (Gao et al., 2018). NFT causes abnormal protein accumulation by downregulating proteasome activity, ultimately leading to neuronal damage (George and Tepe, 2021). Tau protein interacts with microglia. Tau protein can stimulate the activation of microglia, change the phenotype of microglia, and promote the overexpression of cytokines. At the same time, activated microglia have the ability to absorb and destroy pathogenic tau protein, but can also help the pathological expansion of tau protein (Asai et al., 2015; Bolós et al., 2016). TREM2 signal transduction in microglia is also related to tau pathology in the brain of AD patients (Laurent et al., 2018). As mentioned above, TREM2 signaling pathway mediates the phagocytosis of microglia to pathological products of AD, including pathogenic tau protein. As confirmed by research, upregulation of TREM2 expression can reduce tau protein hyperphosphorylation in AD model mice (Peng et al., 2023). Other studies have shown that TREM2 can inhibit the infiltration of tau protein into the hippocampus, which is important for preventing synaptic loss (Zhu et al., 2022). In addition, microglia can also internalize through the interaction between chemokine receptor CX3CR1 and extracellular tau protein (Chidambaram et al., 2020). Contrary to phagocytosis, microglia can induce tau protein hyperphosphorylation by secreting inflammatory factors, driving the pathological development of tau protein (Wang C, et al., 2022). Recent research shows that tau protein can also activate NF-κB pathway in microglia enhances TLR signal transduction, while the activation of NF-κB pathway can also mediate cellular phagocytosis and enhance the clearance of tua protein (Qin et al., 2016). In addition, the tau proteasome and autophagy system mediate the decomposition of tau protein, and there are indications that both of these clearance mechanisms are damaged to a certain extent in AD (Tang et al., 2019).

### 3.3 Synaptic deletion and microglia

Synapse, as a structure connecting neurons, is a bridge for information transmission in the CNS. Synaptic plasticity is the basis for normal synaptic function and has important contributions to learning and memory (Nan et al., 2017). The hypothesis of pathological synaptic loss suggests that the main causes of memory impairment and cognitive decline in AD are not only neuronal loss but also Aβ The accumulation of plaque and NFT, synaptic loss, and synaptic dysfunction are also significant factors (Ziegler Waldkirch and Meyer, 2018). Aβ Plaque and NFT can induce inflammatory activation of microglia and complement cascade dependent synaptic clearance, thereby promoting AD pathology (Li et al., 2022). Aβ activates microglia to release pro-inflammatory factors, including TNF-α, and disrupts the balance of synaptic connections (Baj and Seth, 2018; Liu, 2022), IL-1β mediates the toxic effects of Aβ on synapses, leading to changes in synaptic plasticity (Batista et al., 2021). In addition, studies have found that the expression of complement C1q and C3 is upregulated in the hippocampus of AD model mice, and through synaptic connection, it leads to an increase in the phagocytosis of synaptic elements by microglia, leading to a decrease in the number of synapses (Xin et al., 2019). Inhibition of C1q, C3, and CR3 can save synaptic loss and cognitive dysfunction in AD model mice, further supporting complement mediated microglial phagocytosis involved in AD synaptic loss (Tang et al., 2019). During the progression of AD, the phagocytic function of microglia is regulated by TREM2. In early AD, TREM2 predominantly mediates the conversion of microglia to a synaptoprotective state and mediates the scavenging of Aβ by microglia, however, in late AD, TREM2 predominantly mediates the overactivation of microglia and the scavenging of synapses (Krasemann et al., 2017). These results suggest that the regulation of synaptic plasticity by microglia may play an opposite role in the early and late stages of AD, which is consistent with the view that microglia play an opposite role in different stages of AD.

### 3.4 Others

In AD, activated microglia can migrate to amyloid plaques to secrete more inflammatory molecules to accelerate tissue damage. The enhanced migration ability of microglia may lead to greater brain damage (Zhong et al., 2018). This migration ability of microglia is related to the high expression of surface pattern recognition receptors (PRRs) such as Toll-like receptors (Chen et al., 2016; Wang C, et al., 2022). In addition, *in vitro* studies revealed that Aβ was able to bind TREM2 with high affinity, activate the TREM2-dependent signaling pathway and promote microglia migration (Zhong et al., 2018). Repressor Element 1 Silencing Transcription Factor (REST) is highly expressed in the nucleus of microglia. REST inhibits the migration of AD microglia by inhibiting the activity of progranulin (PGRN; Yu et al., 2020).

Other studies suggest that cholesterol-mediated regulation of microglia function may be directly involved in AD pathology, that abnormal cholesterol metabolism is associated with Aβ deposition, and that the ability of microglia to degrade Aβ is dependent on their ability to excrete cholesterol (Dai et al., 2022). High cholesterol concentrations lead to microglial activation, impair the ability of microglia to clear Aβ, and increase microglial inflammatory signaling and ROS production, all of which further contribute to the accumulation of Aβ and induce a pro-inflammatory environment that leads to neurodegeneration (Muñoz Herrera and Zivkovic, 2022). The isoform of Apolipoprotein E (APOE), is known to be associated with an increased risk of AD, and a reduced ability of microglia to excrete cholesterol was observed in microglia expressing APOE4 (Iannucci et al., 2021).

## 4 Mechanism of acupuncture regulating the treatment of AD with microglia

### 4.1 The effect of acupuncture on the inflammatory response

One of the anti-inflammatory mechanisms of acupuncture is to stimulate the connective tissue at the acupoints to deform and induce the secretion of a variety of chemicals, affecting the state of inflammatory cells or immune signaling pathways and regulating the release of cytokines (Li N, et al., 2021). Secondly, it stimulates nerve endings directly or indirectly through the microenvironment, transmits information to the spinal cord, brainstem or hypothalamus, and the brain, the highest center of the human body, integrates the information and gives anti-inflammatory instructions (Zhang and Liu, 2018), And microglia, as a major participant in neuroinflammation, is one of the main targets of acupuncture to regulate neuroinflammation. Acupuncture is able to inhibit the expression of inflammatory factors and promote the release of anti-inflammatory factors by modulating the polarization state of microglia as well as inflammatory signaling pathways and other pathways. The basic research on the modulation of microglia neuroinflammation by acupuncture is shown in Table 1.

**Table 1:** Summary of experiments on acupuncture regulating neuroinflammation in the treatment of AD.

AD Models	Treatment	Acupoints	Target changes		References
			Raise	Lower	
SD rats	EA	Baihui (GV 20), Zusanli (ST 36)		NLRP3, Caspase-1, IL-1β	Chuan et al. (2020a)
5xFAD mice	EA	Zusanli (ST 36)		NLRP3, Caspase-1, IL-1β,IL-18	Ni et al. (2023)
APP/PS1 mice	EA	Baihui (GV 20), Yintang (GV 29)		P2X7R, NLRP3, ASC, Caspase-1, IL-1β	Yu et al. (2023)
SAMP8 mice	MA	Baihui (GV 20), Yintang (CV 29), Shuigou (GV26)		NLRP3, ASC, Caspase-1, and IL-1β	Lin et al. (2023)
SAMP8 mice	EA	Baihui (GV 20), Yintang (CV 29), Renzhong (EX-HN3)		NLRP3, ASC, Caspase-1, and IL-1β	Ding et al. (2017)
5xFAD mice	EA	Benshen (GB 13), Shenting (DU 24)	TFEB, TFE3	NLRP3, IL-1β	Jing (2016)
5xFAD mice	EA	Zusanli (ST 36)		NLRP3, caspase-1, ASC, IL-1β, IL-18	Ni et al. (2023)
PS cDKO mice	EA	Baihui(GV 20), Shenting(DU 24)		NLRP3, ASC, Caspase-1, IL-1β, IL-18	Li K, et al. (2021)
SAMP8 mice	EA	Baihui (GV 20), Yintang (GV 29)	TREM2, DAP12		Jing et al. (2016)
APP/PS1 mice	EA	Baihui (GV 20), Yintang (GV 29), Shuigou (GV 26)	TREM2, DAP12, PI3K, Akt, PLC-y	TNF-α, IL-1β, IL-6, IL-12	Jin (2018)
SD rats	EA	Baihui(GV 20), Zusanli(ST 36)		TLR4, NF-κB P65, IL-1β, IL-6, TNF-α	Chuan et al. (2020b)
SD rats	MA	Baihui(GV 20), Shenshu(BL 23)		JAK2, STAT3	Liu et al. (2019)
SD rats	MA	Baihui(GV 20), Shenshu(BL 23)		JAK2, STAT3, IL-1β, IL-6	Qin et al. (2020)
SAMP8 mice	EA	Yintang(GV 29), Yingxiang(LI 20)	SYN, PSD95	GSK-3β, IL-1β, TNF-α	Yuan et al. (2021)
SAMP8 mice	EA	Yintang(GV 29), Yingxiang(LI 20)		p38MAPK	Wang et al. (2020b)
APP/PS1 mice	EA	Baihui(GV 20), Yintang(GV 29), Renzhong(EX-HN3)		COX-2, TGF-β1	Qiujie (2018)
APP/PS1 mice	EA	Baihui(GV 20), Renzhong(EX-HN3)		GFAP, IBA-1, IL-lβ, IL-6, TNF-α	Wang L M, et al. (2020)
SD rats	EA	Baihui(GV 20)	Arg1	iNOS, IL-1β, TNF-α	Xie Lusang and Qiaofeng (2018)
APP/PS1 mice	EA	Baihui(GV 20), Renzhong(EX-HN3), Yintang(GV 29)	IL-10	HMGB1	Jianghao (2018)
APP/PS1 mice	EA	Baihui(GV 20), Shenting(DU 24)	CD206, Arg1	iNOS, IL-1β	Sheng (2019); Li L, et al. (2020)
5xFAD mice	EA	Taixi(KI 3)		CD11b, GFAP, COX2,HO-1, transferrin and Bax	Cai et al. (2019)
SAMP8 mice	EA	Yingxiang(LI 20), Yintang(GV 29)		HMGB1, TLR4, RAGE, NADPH	Wang et al. (2023)

#### 4.1.1 NLRP3 inflammatory corpuscles

NLRP3 inflammatory body is a polymeric protein complex composed of NOD-like receptor thermal protein domain associated protein 3(NLRP3), apoptosis-associated speck-like protein containing CARD (ASC), and Caspase-1 precursor protein (Li and Hongju, 2018), plays a key role in various neurodegenerative diseases such as AD. NLRP3 protein regulates IL-1β by activating downstream ASC proteins, thereby activating the Caspase-1 molecular platform. “The maturation and secretion of pro-inflammatory cytokines such as IL-1β also mediate programmed cell death, which is known as’ pyrolytic death (Moonen et al., 2023). The chronic inflammatory response of AD is closely related to the excessive activation of inflammatory bodies. An autopsy of brain tissue from a patient with severe AD reported NLRP3, Casepase-1, and their downstream substance IL-1β in the patient’s brain tissue. The expression of NLRP3 inflammasomes is increased, which confirms the activated state of NLRP3 inflammasomes in AD (Saresella et al., 2016). Studies have found that the activation of NLRP3 inflammatory bodies in AD can be inhibited by intracellular Aβ Induce the release of cathepsin B (Park et al., 2019). It has also been shown that up-regulation of Caspase-1 and NLRP3 expression promotes brain damage in AD model mice (Qiu et al., 2020), and that reduced IL-1β and Caspase-1 concentrations contribute to Aβ clearance (Aricioglu et al., 2019). Intracellular activated NLRP3 inflammatory vesicles observed in AD model mice promote activation of microglia M1 phenotype, leading to Aβ accumulation and more severe cognitive impairment (Sharma and Kanneganti, 2016), while the inactivated microglia of NLRP3 inflammatory bodies are biased toward the M2 phenotype, playing a neuroprotective role in regulating neuronal inflammation (Zhang et al., 2020). In conclusion, activation of NLRP3 inflammatory vesicles in microglia by Aβ has the potential to be a new target for the treatment of inflammatory responses in AD (Stancu et al., 2019).

Research shows that electroacupuncture pretreatment of Baihui and Zusanli points in AD model rats can inhibit the expression of NLRP3 inflammatory body related protein and microglia activation, reduce Aβ sedimentation, inhibit the release of inflammatory factors, thereby reducing the inflammatory response of the central nervous system(Chuan et al., 2020a; Ni et al., 2023). Acupuncture at Baihui and Yintang points in AD model mice purinergic 2×7 receptor (P2X7R; Yu et al., 2023) can cause NLRP3, ASC, Caspase-1, and IL-1β in hippocampal microglia (Li K, et al., 2021; Lin et al., 2023; Ni et al., 2023). The expression of proinflammatory proteins is effectively reduced, and the inflammatory response of the central nervous system is attenuated or inhibited to a certain extent. Therefore, it plays a protective role in neurons (Jing, 2016; Ding et al., 2017). In summary, it can be inferred that inhibiting the activation of NLRP3 inflammatory bodies is one of the ways in which acupuncture exerts its inhibitory effect on the inflammatory response of AD.

#### 4.1.2 Inflammation related pathways

1. TREM2 pathway: Genome-wide association study reveals genetic variants highly expressed in microglia for AD pathogenesis, with rare missense mutations in TREM2 increasing the risk of AD nearly threefold (Ayyubova, 2023). Aβ inhibits microglia activation as well as enhances microglia phagocytosis through activation of the microglia receptor TREM2 protein (Basha et al., 2023). Among them, the role of TREM2 protein in mediating the inflammatory activation of microglia is to activate the downstream DAP12 protein (DAP12), thereby regulating multiple signal pathways such as the PI3K/AKT signal pathway and the FoxO5a signal pathway (Wang et al., 2020a; Yaping, 2020). Studies have shown that acupuncture at Baihui and Yintang points can inhibit the activation of microglia in the hippocampus of mice with AD model, increase the expression of TREM2 and DAP12 proteins, reduce the release of cellular inflammatory factors, improve spatial learning and memory ability, and improve the morphology and structure of neurons in the hippocampus (Jing et al., 2016). On the basis of acupuncture at Baihui and Yintang points with the method of “Tongdu Qishen,” increasing the Shuigou point can also increase the expression level of proteins such as TREM2 and DAP12 in microglia, and inhibit the release of the inflammatory product TNF-α, IL-1β, IL-6 and IL-12 in AD pathology, and inhibition of chronic inflammatory reactions in the brain, thereby improving the spatial learning and memory abilities of AD model mice (Jin, 2018). In summary, electroacupuncture helps microglia play an anti-inflammatory role in protecting neurons by upregulating the expression of TREM2 in the hippocampus and inhibiting the expression of proinflammatory factors (Li Y, et al., 2020). The above is the role of acupuncture in regulating TREM2 mediated inflammation, and the phagocytosis will be described in detail in the following phagocytosis related pathways.
2. TLR4/NF-κB-pathway: Toll-like receptor 4 (TLR4) is an important pattern recognition receptor in the Toll-like receptor family. It is mainly expressed in microglia in the brain and can specifically bind to stimulators, thereby activating downstream Nuclear Factor kappa-B (NF-κB), up-regulating the expression of proinflammatory factors (Garcia Bueno et al., 2016; Leitner et al., 2019). TLR4/NF-κB signal pathway regulates the occurrence and development of inflammation and is closely related to immune mechanisms (Na et al., 2015). Similarly, during the inflammatory reaction of AD, the TLR4/NF-κB signal pathway plays an important role. Research shows that Aβ Ability to activate TLRs/NF-κB signal pathway, regulating its downstream inflammatory factor IL-1β, IL-6, TNF-α (Leitner et al., 2019). Studies have shown that pre acupuncture at Baihui and Zusanli in AD model rats can effectively downregulate TLR4 and NF-κB Expression of P65 protein reduces IL-1β, IL-6, TNF-α (Chuan et al., 2020b), which suggests that pre acupuncture can inhibit the release of TLR4/NF-κB in the brain tissue of AD model rats, The effective regulation of signal pathway and inflammatory mediator release may be one of the mechanisms by which it can alleviate central inflammatory reactions and improve spatial learning and memory abilities in AD model rats. Other studies have used the “Tongdu Qishen” method to electroacupuncture Baihui, Yintang, and Shuigou points in AD model mice. The results show that acupuncture reduces the co expression of microglia and their TLR4 pattern recognition receptors, and can also affect the downstream proteins of their signal pathways, including Myeloid Differentiation Factor 88 (MyD88), and transforming growth factor β TGF beta activated kinase 1 (TAK1), tumor necrosis factor receptor associated factor 6 (TRAF6), and NF-κB. The expression of oxidative stress product iNOS and costimulatory molecule CD40 has a significant negative regulatory effect. One of the mechanisms by which acupuncture can improve learning and memory impairment in AD model mice may be through inhibiting the activation of microglia, thereby slowing down neuronal apoptosis (Lu, 2019).
3. JAK/STAT pathway: The tyrosine protein kinase signal transducer and activator of transcription 3 (JAK2/STAT3) signal pathway mainly affects AD production from two aspects: regulating central inflammatory response and interfering with the survival of glial cells (Qian et al., 2015). STAT3 is a downstream signaling mediator of IL-10 and plays an anti-inflammatory role in many pathophysiological processes. Studies have confirmed that the expression level of STAT3 in hippocampal neurons of both AD model mice and AD patients decreases (Liu et al., 2022). Studies show that Aβ downregulates the JAK2/STAT3 signaling pathway in microglia and inhibits inflammatory responses (Zhang et al., 2014). Acupuncture at Baihui and Shenshu has a significant inhibitory effect on the abnormal activation of JAK2/STAT3 signal pathway in the cerebral cortex of AD model rats, with a decrease in the content of inflammatory cytokines, and repair of neuronal structure and function (Liu et al., 2019; Qin et al., 2020). It is inferred that acupuncture regulating the JAK2/STAT3 signaling pathway in the brain tissue of AD model rats may be one of the mechanisms by which acupuncture inhibits the inflammatory response of the central nervous system.
4. PI3K/Akt pathway: The phosphatidylinositol 3-kinase/protein kinase B (PI3K/Akt) signal pathway has the functions of regulating cell proliferation, differentiation, metabolism, and anti apoptosis, and is an important pathway for membrane receptor signal transduction into cells (Liyuan et al., 2020). Activation of PI3K/Akt upregulates the expression of postsynaptic density protein 95, synaptophysin, and GAP43, protects hippocampal synaptic plasticity, prevents neuronal cell death in AD, and has neuroprotective effects on AD model rats (Wang et al., 2021). Previous studies have shown that the PI3K/Akt signaling pathway plays an anti-inflammatory role in microglia and increases AKT signaling, thereby inhibiting the release of proinflammatory cytokines (Tu et al., 2016). It was also found that electroacupuncture at Baihui, Yintang and Shuigou points with the method of “Tongdu Qishen” could increase the expression level of key proteins of microglia activation pathway, such as PI3K and Akt, and inhibit the expression of inflammatory products of microglia activation, such as TNF-α, IL-lβ, IL-6 and IL-12, and inhibit the chronic inflammatory response in the brain. Inhibited the expression of microglia activation inflammatory products such as TNF-α, IL-lβ, IL-6 and IL-12, and suppressed the chronic inflammatory response in the brain, which in turn improved the spatial learning and memory ability of AD model mice (Jin, 2018). Research has found that “olfactory three needles” intervention in AD model mice could regulate Aβ deposition by strengthening the PI3K/Akt signaling pathway and inhibit the expression of its downstream key protein kinase glycogen synthase kinase-3β (GSK-3β), thereby increasing the expression of synapse-related molecules such as SYN and PSD95, inhibiting inflammatory cytokines IL-1β, TNF-α and neuronal apoptosis, and repairing the morphological and structural damage of hippocampal synapses. SYN and PSD95 and other synapse-related molecules, inhibit inflammatory cytokines IL-1β, TNF-α and neuronal apoptosis, repair hippocampal synaptic morphological and structural damage, and improve hippocampal synaptic plasticity, thereby improving cognitive function and learning and memory ability in AD (Yuan et al., 2021). This indicates that acupuncture can inhibit inflammation and improve synaptic function by activating the PI3K/Akt signaling pathway to improve AD symptoms.
5. Other: Acupuncture of Yin Tang and Ying Xiang points enhances spatial learning and memory by inhibiting phosphorylation of p38 mitogen-activated protein kinase (p38MAPK) in microglia and suppressing overactivation of microglia to reduce neuroinflammatory responses and neurotoxicity of Aβ and to promote synaptic regeneration, and Improvement of cognitive ability in AD model mice (Wang et al., 2020b). It was found that electroacupuncture at Baihui, Yintang and Renzhong points in mice with the “Tongdu Qishen” method could reduce the expression of inflammatory mediators Cyclooxygenase-2 (COX-2) and Transforming Growth Factor β1 (TGF-β1), thereby reducing Aβ deposition and inhibiting microglial cell activation. TGF-β1, thereby reducing Aβ deposition and inhibiting microglia activation (Qiujie, 2018). Other studies have shown that electroacupuncture at Shangxing and Baihuanshu points in AD model mice can inhibit the activation of microglia, reduce glial fibrillary acidic protein (GFAP), ionized calcium binding receptor molecule-1 (IBA-1), and IL-lβ in the hippocampus、IL-6 and TNF-α expression of inflammatory factors and Aβ (Cai et al., 2019; Wang L M, et al., 2020), may help to improve recognition memory and slow the development of AD.

#### 4.1.3 Microglia polarization

Studies have found that acupuncture at Baihui point can promote the polarization of microglia toward M2 type in AD model rats, increase the expression of Arg1 protein in hippocampus, inhibit the production of M1 type microglia, and reduce iNOS protein and IL-1β And TNF-α “Expression of β - lactamase (Xie Lusang and Qiaofeng, 2018). Other studies have found that acupuncture at Baihui, Renzhong, and Yintang points with the “Tongdu Qishen” method can maintain the balance of two polarization states of microglia in AD model mice, which is achieved by inhibiting the expression of M1 type polarizing inflammatory factor high mobility group protein 1 (HMGB1) and promoting M2 type polarizing inflammatory factor IL-10 (Jianghao, 2018; Wang et al., 2023). Electroacupuncture at Baihui and Shenting points in AD model mice can regulate the polarization of microglia and inhibit M1 markers iNOS and IL-1β and increases the expression of M2 markers CD206 and Arg1 in these regions, thereby playing a neuroimmunomodulatory role and reducing the expression of Aβ “Plaque aggregation affects the functional activity of its hippocampus, thereby improving the learning and memory function of AD (Sheng, 2019; Li L, et al., 2020). Therefore, regulating the polarization state of microglia is one of the mechanisms of acupuncture treatment for AD.

### 4.2 Effect of acupuncture on phagocytosis of microglia

Acupuncture may indirectly regulate the function of microglia by affecting relevant molecular mechanisms, influencing the expression of cytokines or receptors related to microglia phagocytosis, and thus regulating the phagocytic activity of microglia. The study suggests that electroacupuncture is supposed to improve the synaptic function of AD mice by decreasing the expression of microglia, which in turn inhibits the activation of the complement system, thus improving learning and memory ability (Hong et al., 2022).

#### 4.2.1 Phagocytosis related pathways

The phagocytic function of microglia refers to the process of recognizing, phagocytosing, and clearing “waste” in brain tissue mediated by receptors to maintain CNS homeostasis (Xue et al., 2022). TREM2 upregulates CCAAT/enhancer binding protein α (C/EBP α) Increase CD36 expression to enhance the sensitivity of microglia to Aβ Phagocytosis, protecting neurons from Aβ Induced cytotoxicity (Kim et al., 2017). Studies have shown that overexpression of TREM2 can enhance the function of microglia in phagocytosis of apoptotic neurons (Zhao et al., 2022), while the deletion of TREM2 prevents the accumulation of microglia around amyloid plaques, leading to barrier defects that limit neuronal damage (Yeh et al., 2017). Compared with healthy individuals, AD patients do exhibit high levels of TREM2 in their cerebrospinal fluid (Smirnov and Galasko, 2022). Studies have found that electroacupuncture at Baihui can upregulate the expression of TREM2 on microglia (Yang et al., 2023), it is speculated that acupuncture may enhance the phagocytosis of microglia through the TREM2 pathway. Although acupuncture can improve the phagocytosis of microglia by upregulating the expression of TREM2, there is currently no research on its application to AD, so further in-depth research is needed. Electroacupuncture at Benshen and Shenting points in AD model mice regulates kinases including AMPK and AKT to activate the transcription factor EB (TFEB) and the transcription factor E3 (TFE3), promoting TFEB/TFE3 mediated Aβ (Lin et al., 2023).

#### 4.2.2 Complement system

The complement system is an important component of the innate immune system, which has the effect of enhancing the ability of phagocytes to clear microorganisms or damaged cells. Microglia exert their phagocytic ability through the role of complements (Chung et al., 2016). The classic activation pathway of the complement system is the activation of C1r, C1s, C4, C2, and C3 initiated by the antigen antibody complex binding initiation protein C1q, forming a cascade of enzymatic reactions between C3 invertase (C4b2a) and C5 invertase (C4b2a3b), which generally plays a role in the late stage of infection. The classic complement cascade mediates the synaptic clearance process in the central nervous system (Presumey et al., 2017), in which complement proteins C1q and C3 are involved in the process of phagocytosis of synapses by microglia (Ricklin et al., 2016). Specifically, during the development of the central nervous system, redundant neuronal synapses express complement protein C1q, which binds to CR3 receptors on the surface of microglia to activate the phagocytosis of microglia to neural synapses (Norris et al., 2018). Inhibition of C1q, C3, or microglial complement receptor CR3 can weaken the phagocytosis of microglia and prevent synaptic loss (Werneburg et al., 2020). Studies have shown that electroacupuncture at Baihui, Dazhu, and Shenshu in AD model mice can inhibit the phagocytosis of complement C1q dependent microglia in the hippocampus, thereby inhibiting the phagocytosis of microglia to neural synapses, and achieving the function of protecting the learning and memory functions of AD model mice (Hong et al., 2022).

#### 4.2.3 Proteasome system

Matrix metalloproteinases (MMPs), also known as matrix proteins, are a family of zinc dependent endopeptidases (de Almeida et al., 2022). Among them, MMP-2 and MMP-9 are expressed in microglia (Lindhout et al., 2021), which is related to the mechanism by which microglia play a scavenging role. MMP-2 and MMP-9 are known to be capable of cutting Aβ Monomers and oligomers, especially MMP-9, can also cut Aβ Fibroblasts and clearing amyloid rich brain plaques increase Aβ Interventions for enzyme degradation have been shown to reduce AD pathology (Loeffler, 2023). Research has found that electroacupuncture at Baihui, Dazhui, Shenshu, Zusanli, and Taixi points can reduce the expression of MMP-2 and MMP-9 in microglia, thereby inhibiting neuronal apoptosis, thereby improving the learning and memory abilities of AD model rats (Zhaofu et al., 2013).

## 5 Discussion

**Figure 2:**
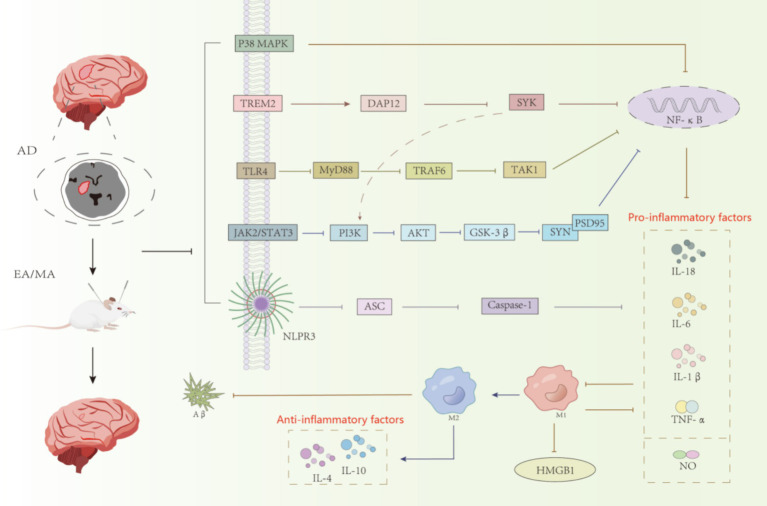
Acupuncture regulates the relevant inflammatory pathways in AD animal models.

Microglia, the main immune executors of the CNS, have a strong influence on neurogenesis through inflammation-mediated and phagocytic capacities (Sierra et al., 2010; Tang and Le, 2016). Activation of microglia in AD is dynamic and complex, ranging from progressive activation to partial activation to complete activation (Li et al., 2022). Once abnormally activated, microglia can play a harmful role in cognitive dysfunction, directly or indirectly damaging neurons (Colonna and Butovsky, 2017). Acupuncture, as one of the important therapeutic methods in traditional Chinese medicine, has a quite definite therapeutic effect on AD. The mechanism of acupuncture regulating the treatment of AD by microglia is mainly divided into two categories. The first aspect was to improve the inflammatory response of CNS. As shown in Figure 2, acupuncture reduced the expression of ASC, Caspase-1, and IL-1β by inhibiting the expression of NLRP3 inflammatory vesicles; acupuncture inhibited the expression of GSK-3β by inhibiting the TREM2 pathway and its downstream DAP12 proteins and the JAK2/STAT3 pathway, which together regulate the PI3K/AKT signaling pathway and inhibit the expression of GSK-3β, which in turn increased SYN and PSD95 and other synapse-related molecules; secondly, acupuncture inhibited the expression of TLR4, decreased the levels of downstream proteins MyD88, TAK1 and TRAF6, and the three pathways of TREM2 and JAK2/STAT3 and TLR4 simultaneously inhibited NF-κB, and finally, it regulated the polarization of microglia from the M1 to the M2 direction, decreased the pro-inflammatory cytokines (IL-1β, TNF-α and IL-6, etc.) and increase the expression of anti-inflammatory cytokines (IL-4 and IL-10, etc.), which leads to the inhibition of CNS inflammatory response and protection of neurons. The second aspect is to regulate the phagocytic activity of microglia. Acupuncture promotes the clearance of abnormally accumulated proteins by promoting TREM2 receptors and regulating the expression of related phagocytosis proteins, inhibiting the complement system C1q and the proteasome system MMP-2 and MMP-9, in order to reduce the neuroinflammatory and neurotoxicity of Aβ (Tang and Le, 2016). These results suggest that electroacupuncture aids neurogenesis by increasing microglia phagocytosis and inhibiting pro-inflammatory processes.

In this review, the animal models included for basic research are mainly three types: SAMP8 mice, APP/PS1 mice, and 5xFAD mice, and the interventions are based on electro-acupuncture, with preferred acupoints Baihui (GV 20) and Yintang (GV 29). By stimulating the acupoints, not only can the local nerve endings be activated to influence the function of other parts of the body through nerve reflexes, but also local blood circulation can be promoted to improve the nutrient supply to the tissues and the elimination of metabolic wastes. According to the literature review, the duration of needle retention is mostly 20–30 min, and the acupuncture cycle is mainly 2–6 weeks. As an intervention means, the biological effect of acupuncture may take some time to appear, and the time window of 2–6 weeks can observe the chronic effects of acupuncture on animal models, including behavioral changes, changes in biochemical indexes, etc., which can provide a reference for the design of clinical treatment protocols. In addition to this, this paper summarizes the targets of action in basic research, biomarkers that can help monitor the effectiveness of acupuncture in treating AD, a better understanding of the mechanism of action of acupuncture in treating AD, and implications for more standardized acupuncture protocols and biopharmaceuticals. It is suggested that early intervention of acupuncture can inhibit excessive inflammatory response and enhance phagocytosis of microglia, and future studies should further explore the compatibility of acupoints, the dose-effect relationship, and the optimal time-volume parameter that affects the effectiveness of acupuncture intervention to guide clinical practice and the development of the discipline.

The mechanism of acupuncture treatment for AD is complex, involving multiple aspects, and there are certain connections among various aspects. There is also a certain correlation between various signal pathways involved in microglia, so different signal pathways are worth further exploration. This study elaborates on the correlation between relevant molecules and pathways involved in the mechanism of acupuncture treatment of microglia in AD, and conducts in-depth research on this aspect to provide more reliable evidence for the mechanism of acupuncture regulation of microglia in the treatment of AD.

## Author contributions

JL: Investigation, Writing – original draft, Writing – review & editing. JZ: Methodology, Supervision, Writing – review & editing. CY: Investigation, Methodology, Writing – original draft. HX: Formal analysis, Resources, Writing – original draft. YG: Formal analysis, Resources, Writing – review & editing. YL: Resources, Supervision, Writing – review & editing. XG: Conceptualization, Supervision, Writing – review & editing.

## Glossary

AD: Alzheimer’s disease
Aβ: amyloid β-protein
CNS: Central Nervous System
Arg1: Arginase-1
iNOS: Inducible nitric oxide synthase
TGF-β: Transforming growth factorβ-1
IGF-1: Insulin-like growth factors
BDNF: Brain-derived neurotrophic factor,
TREM2: Triggering Receptor Expressed On Myeloid Cells 2
SYK: Spleen tyrosine kinase
TLR: Toll-like receptor
RAGE: Receptor For Advanced Glycation End
CR3: complement receptor 3
APP: Amyloid precursor protein
NFT: Neurofibrillar tangle
PRR: Pattern Recognition Receptor
REST: Repressor Element 1 Silencing Transcription Factor
NLRP3: NOD-like receptor thermal protein domain associated protein 3
JAK2/STAT3: Januskinase 2-signal transducer and activator of transcription 3
PI3K/Akt: phosphoinositide 3-kinase/protein kinase B
p38MAPK: p38 mitogen-activated protein kinase
MMPs: Matrix metalloproteinases
